# Correlation between rCBV Delineation Similarity and Overall Survival in a Prospective Cohort of High-Grade Gliomas Patients: The Hidden Value of Multimodal MRI?

**DOI:** 10.3390/biomedicines12040789

**Published:** 2024-04-03

**Authors:** Amina Latreche, Gurvan Dissaux, Solène Querellou, Doria Mazouz Fatmi, François Lucia, Anais Bordron, Alicia Vu, Ruben Touati, Victor Nguyen, Mohamed Hamya, Brieg Dissaux, Vincent Bourbonne

**Affiliations:** 1 Radiation Oncology Department, University Hospital, 29200 Brest, France; amina.latreche@chu-brest.fr (A.L.); gurvan.dissaux@chu-brest.fr (G.D.); francois.lucia@chu-brest.fr (F.L.); anais.bordron@chu-brest.fr (A.B.); alicia.vu@chu-brest.fr (A.V.); victor.nguyen@chu-brest.fr (V.N.); mohamed.hamya@chu-brest.fr (M.H.); 2 Nuclear Medicine Department, University Hospital, 29200 Brest, France; solene.querellou@chu-brest.fr; 3 Groupe d’Etude de la Thrombose Occidentale GETBO (INSERM UMR 1304), Université de Bretagne Occidentale, 29200 Brest, France; 4 Radiology Department, University Hospital, 29200 Brest, France; doria-m@hotmail.fr; 5 LaTIM UMR 1101, INSERM, Université de Bretagne Occidentale, 29200 Brest, France

**Keywords:** high-grade gliomas, radiotherapy, delineation, MRI, PET-FET, rCBV, glioblastoma, radiotherapy, magnetic resonance imaging, [^18^F]-FET PET/CT

## Abstract

Purpose: The accuracy of target delineation in radiation treatment planning of high-grade gliomas (HGGs) is crucial to achieve high tumor control, while minimizing treatment-related toxicity. Magnetic resonance imaging (MRI) represents the standard imaging modality for delineation of gliomas with inherent limitations in accurately determining the microscopic extent of tumors. The purpose of this study was to assess the survival impact of multi-observer delineation variability of multiparametric MRI (mpMRI) and [^18^F]-FET PET/CT. Materials and Methods: Thirty prospectively included patients with histologically confirmed HGGs underwent a PET/CT and mpMRI including diffusion-weighted imaging (DWI: b0, b1000, ADC), contrast-enhanced T1-weighted imaging (T1-Gado), T2-weighted fluid-attenuated inversion recovery (T2Flair), and perfusion-weighted imaging with computation of relative cerebral blood volume (rCBV) and K2 maps. Nine radiation oncologists delineated the PET/CT and MRI sequences. Spatial similarity (Dice similarity coefficient: DSC) was calculated between the readers for each sequence. Impact of the DSC on progression-free survival (PFS) and overall survival (OS) was assessed using Kaplan–Meier curves and the log-rank test. Results: The highest DSC mean values were reached for morphological sequences, ranging from 0.71 +/− 0.18 to 0.84 +/− 0.09 for T2Flair and T1Gado, respectively, while metabolic volumes defined by PET/CT achieved a mean DSC of 0.75 +/− 0.11. rCBV variability (mean DSC0.32 +/− 0.20) significantly impacted PFS (*p* = 0.02) and OS (*p* = 0.002). Conclusions: Our data suggest that the T1-Gado and T2Flair sequences were the most reproducible sequences, followed by PET/CT. Reproducibility for functional sequences was low, but rCBV inter-reader similarity significantly impacted PFS and OS.

## 1. Introduction

High-grade gliomas (HGGs) are the most common primary malignant brain tumors arising from glial cells [1]. Treatment usually involves surgical resection when feasible, followed by chemoradiotherapy (CRT) with concurrent temozolomide and adjuvant temozolomide [2].

Relapse of HGGs is unfortunately inescapable [1], the majority of tumor recurrences after radiotherapy occurring within the treated volume [3], thus highlighting the need for complementary imaging assessment for improved target volume delineation.

The tumor definition for radiotherapy treatment planning is based on morphological imaging techniques, mostly on contrast-enhanced T1-weighted magnetic resonance imaging (MRI) [4]. This morphological characterization could under-evaluate the extension, in comparison with functional and metabolic assessments, and therefore the accuracy of treatment [5].

HGGs are characterized by an extensive microvascular proliferation with elevated permeability in their center or in surrounding tissue [6], making their extension limits difficult to define. Dynamic susceptibility contrast brain MRI evaluates several aspects of brain vasculature by assessing different parameters such as relative cerebral blood volume (rCBV) and permeability. It could provide additional information compared to conventional MRI [7] to allow a more robust characterization of the peritumoral area. Moreover, diffusion-weighted imaging (DWI) and apparent diffusion coefficient (ADC) maps illustrate restricted diffusion of water molecules in the cellular microenvironment [8]. The degree of diffusion restriction correlates directly, while ADC values are inversely correlated with increased tissue cellularity [9,10]. Identifying these areas of restricted diffusion may serve to focus treatment on active tumor tissue. The use of additional information from biological imaging such as [^18^F]FET (fluoroethyl-l-tyrosine) positron emission tomography/computed tomography (PET/CT) provides a molecular evaluation of malignant abnormalities [11] leading to a more refined analysis of tumoral behavior. A number of studies have proven the clinical value of amino acid tracers, such as [^18^F]FET PET/CT, to determine the extent of cerebral gliomas for treatment planning [5], the detection of tumor recurrence, and the estimation of prognosis [12,13,14].

Fusing data extracted from the multiparametric perfusion MRI and PET/CT could help in the definition of target volumes and possibly improve patients’ outcomes. Using prospectively acquired data on 30 patients, Dissaux et al. demonstrated the greater reproducibility of morphological MRI sequences and 18F-FET PET/CT over functional MRI sequences [15]. However, the possible impact of delineation variability on survival endpoints was not analyzed [15,16,17,18]. Using volumes defined by spectroscopy or morphological MRI sequences or even PET/CT, several teams have studied the impact of dose escalation. When pooling the data, a nonsignificant survival benefit was found when comparing dose-escalated chemo-radiotherapy to usual chemo-radiotherapy [19]. The definition of target volumes for dose escalation is debatable. An imaging modality on which delineation variability impacts survival could be meaningful in a dose-escalation strategy.

The aim of this study is to assess the reproducibility and complementarity of morphological, functional, and metabolic volumes provided by multiparametric MRI (mpMRI) and PET/CT, as well as to explore the potential survival impact of the inter-reader variability in HGG patients treated with CRT.

## 2. Materials and Methods

The IMAGG study [7] is a prospective monocentric study conducted at a single institution (University Hospital of Brest). It was approved by the institutional review board of the University Hospital of Brest (N°2016.CE14) on 31 March 2016 and registered in the ClinicalTrial.gov registry (NCT03370926). Each patient provided written consent to participate in the study after being fully informed about its details. The full title of the IMAGG study is “FET-PET and Multiparametric MRI for high-grade glioma patients undergoing radiotherapy”.

Additional data pertaining to this specific study were retrospectively collected from the IMAGG cohort.

### 2.1. Patient Population

The eligible patients were older than 18 years and had a histologically proven HGG (grade 3 or 4 according to 2016 World Health Organization) and a performance index score ≤ 2. The exclusion criteria were pregnant or breastfeeding women, contraindications to MRI and/or 18 F-FET PET/CT, and a history of brain radiotherapy. Patients with a recurrent HGG were also excluded.

We collected clinical data including age, sex, date of diagnosis, progression-free survival (PFS), and overall survival (OS). PFS refers to the time from initiation of treatment to the occurrence of disease progression (defined either clinically or radiologically). OS was defined as the duration from initiation of treatment to the occurrence of death. Patients who did not experience an event were censored at the time of the last known visit.

### 2.2. Imaging Protocol

The study protocol specified that the interval between histological confirmation and CRT should not exceed one month and the time between MRI and 18 F-FET PET/CT should not exceed 14 days.

#### 2.2.1. 1/. Magnetic Resonance Imaging

MRIs were performed using a 1.5 T Optima MRI scanner (General Electric Medical Systems, Chicago, IL, USA) or a 1.5 T Magnetom Avanto Fit (Siemens healthineers, Knoxville, TN, USA) or a 3T Achieva dStream MRI (Philips Healthcare, Best, The Netherlands). Succinctly, diffusion-weighted imaging (DWI) with b0 and b1000 was performed. Apparent diffusion coefficient (ADC) maps were calculated. A T2-weighted fluid-attenuated inversion recovery (T2FLAIR) sequence was performed. A T1-weighted MRI (T1Gado) scan was acquired after injection of a standard dose of contrast agent (Gd-DTPA; 0.1 mmol/kg body weight). Perfusion-weighted imaging (PWI) was performed with dynamic susceptibility contrast MR perfusion. Maps depicting relative cerebral blood volume adjusted for contrast leakage (rCBV) and a map estimating permeability (k2) were produced from perfusion-weighted imaging (PWI) data using Olea software version 3.0 from Olea Medical, located in La Ciotat, France [20,21]. The Supplementary Table S1 provides further details on MRI acquisition parameters for the mainly used MRI scanners.

#### 2.2.2. 2/18. F-FET PET/CT

PET imaging was performed on a Biograph mCT PET/CT system (Siemens, Siemens Healthineers, Knoxville, TN, USA). For attenuation correction, a low-dose CT scan was performed without iodine contrast. CT acquisition parameters were 16 × 1.2 mm pitch 0.55 with automatic kVp and mAs modulation. CT reconstruction parameters were as follows: slice thickness 3/3 mm, convolution kernel H31s, and field of view 500 mm for attenuation correction, and slice thickness 2/1.2 mm, convolution kernel J30s, safire 3, and field of view 300 mm for reading. Following the CT examination, the imaging acquisition was focused on the head and involved a 40-min dynamic scan following the intravenous injection of 3 MBq/kg. PET dynamic reconstructions were performed with 10 × 4 min frames, and the reconstruction algorithm was 3DOSEM + TOF + PSF (TrueX) with 200^2^ matrix, 2 iterations, 21 subsets, and gaussian post filter 2 mm. A single static 18F-FET PET frame was obtained by sum in 20–40 min.

All patients fasted for at least 4 h before PET/CT, as per the European Association of Nuclear Medicine guideline for brain tumor imaging using labeled amino-acid analogues [22].

### 2.3. Delineation Protocol

The delineation of target volumes was carried out independently and manually by nine radiation oncologists with varying levels of proficiency in interpreting 18F-FET PET/CT and MRI scans, spanning from residents (with 2 to 5 years of training) to attending physicians (with 1 to 5 years of experience). The delineation process and subsequent data analysis were conducted between September 2021 and July 2022 to evaluate interobserver agreement for both MRI and 18F-FET PET/CT imaging modalities.

This work was performed on MIM Maestro^®^ v7.1.4 software (MiM software Inc., Cleveland, OH, USA). Delineation procedures were conducted without access to initial interpretations, clinical data, or imaging findings. Prior to delineation, a preliminary briefing session was held. For morphological sequences such as T1Gado and T2Flair, segmentation encompassed the entire lesion, including areas of central tumor necrosis or hemorrhage. Conversely, for functional sequences like rCBV, K2, DWI (b1000), and ADC, delineation focused solely on signal abnormalities, including hypersignals indicative of neo-angiogenesis in rCBV, K2, and DWI, as well as hyposignals in ADC. Physicians had the option to refer to morphological sequences during delineation. A gross tumor volume (GTV) was established for each sequence, resulting in six distinct segmentations per reader per patient.

The volume delineation from 18F-FET PET was determined through three-dimensional automatic segmentation using a tumor-to-brain ratio (TBR) of ≥1.6. This threshold is based on a biopsy-controlled study in cerebral gliomas, which found that a lesion-to-brain ratio of 1.6 best distinguishes tumoral from peritumoral tissue [5]. Normal contralateral uptake corresponding to background activity was defined as an area of normal brain tissue, including white and gray matter, on the contralateral hemisphere. It was delineated by drawing a crescent-shaped volume of interest (VOI), referred to as a “banana”, resulting from the summation of 6 subsequent regions of interest (ROIs) 20–25 mm in diameter [23]. For the final step of PET/CT analysis, each physician had the option to refine the automatic segmentation by excluding physiological uptake from the skin or blood vessels.

To limit confusion bias, a specific workflow was designed in MIM displaying each patient’s MRI sequences in this following order: T1Gado, T2Flair, T1Gado + Flair, rCBV, K2, DWI (b1000), and ADC. After rigid registration between the T1Gado MRI sequence and the brain CT, the reader would then move on to PET. The registration was previously assessed by B.D and V.B and was the same for all readers. After performing a segmentation, and to the exception of T1Gado + Flair, the next MRI (or PET) sequence would be displayed shadowing the previous one. This choice was made so that each segmentation would best reflect the information provided by the specific sequence.

### 2.4. Spatial Correlation and Overall Survival Impact

The most appropriate way to carry out the comparison of a segmentation to a group of physician’s segmentations remains complex. In this study, we used different approaches to compare segmentations by assessing the agreement of volume delineated by each physician and comparing them with each other but also by comparing them to a calculated true segmentation [24]. We used two algorithms: simultaneous truth and performance level estimation (STAPLE) and the majority voting rule (MVR). STAPLE takes a collection of segmentations of an image, and computes simultaneously a probabilistic estimate of the true segmentation. Majority voting rule assigns a voxel to the highest class to which at least half of the raters agree on [25].

As a measure of spatial correlation between MRI-based and PET-based volumes, the Dice similarity coefficient (DSC) was calculated [26]. DSC was also used in order to analyze the complementarity of data generated from each sequence of mpMRI and 18F-FET PET. The value ranges between 0, if the volumes are completely disjointed, and 1, if the volumes match perfectly in size, shape, and localization [27]. DSC can be divided into five classes based on its value from 0 to 0.2, 0.2 to 0.4, 0.4 to 0.6, 0.6 to 0.8, and finally, 0.8 to 1 (classes 0, 1, 2, 3, and 4, respectively). Of note, a DSC threshold of more than 0.6–0.7 is often considered as sufficient to characterize the contours as reproducible [28,29]. Descriptive statistics were presented as mean with standard deviation and confidence interval. Mean DSCs by sequence and patient were compared using the Wilcoxon signed-rank test.

Impact of inter-reader variability on PFS and OS was also assessed using Cox regression analysis for both univariate and multivariate analysis, with only the features with a *p* < 0.20 being processed to the multivariate analysis. Surgery, tumor grade methylation of the MGMT (O^6^-methylguanine-DNA-methyltransferase) promoter, and IDH (isocitrate dehydrogenase) mutation status were considered as categorical variables, such as surgery, which was scored as 0 (no surgery) or 1 (surgery: partial or complete excision).

If a DSC score was retained after univariate analysis, mean tumor volume of the corresponding sequence was also analyzed as a continuous variable in the multivariate analysis.

Statistical analysis was performed using Medcalc 15.8.

## 3. Results

From the 30 prospectively included patients, 18FET-PET/CT was available for 29 patients, whereas rCBV and K2 sequences were analyzable for 27 patients due to agent injection issues. All other MRI sequences (T1Gado, T2Flair, b1000, and ADC) were available for analysis.

Patient and tumor characteristics are described in Table 1. Median age was 63 years, with a majority of males (67%). Histologic grade of gliomas was distributed as follows: 4 grade III (13%) and 26 grade IV (87%). Only 8 patients received a complete surgical resection. The median OS was 15 months (IC95: 10–21 months), and the median PFS was 4 months (IC95: 1–8 months).

The highest value for the inter-reader concordance was achieved for morphological sequences. The mean DSC between physicians was 0.84 +/− 0.09 and 0.71 +/− 0.18 for T1Gado and T2Flair, respectively (Table 2).

Metabolic sequences represented by 18FET-PET/CT volume was reproducible with a mean DSC of 0.75 +/− 0.11. Similar results were obtained when comparing the reader-based volumes to MVR or STAPLE volumes as shown in Table 2.

**Figure 1:**
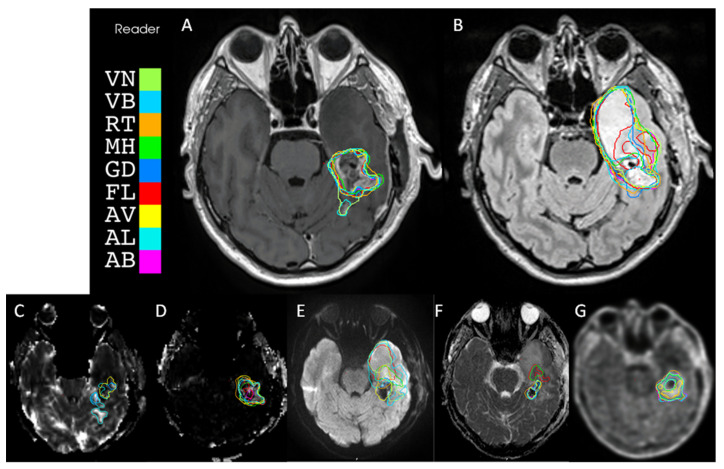
Volume target variability between readers for each sequence. (**A**). T1-weighted imaging (T1Gado). (**B**). T2-weighted fluid-attenuated inversion recovery (T2Flair). (**C**). Relative cerebral blood volume (rCBV) corrected for contrast leakage shows. (**D**). Permeability estimation map (k2). (**E**). Diffusion-weighted imaging (DWI b1000). (**F**). Apparent diffusion coefficient (ADC) map. (**G**). 18F-FET PET/CT.

Regarding the inter-sequence comparison, all sequences were compared to others, using their mean DSC and associated standard deviation. T1Gado appeared to be significantly more replicable than all other segmentations, whereas functional volumes were significantly less reproducible than morphological and metabolic volumes, as shown in Supplementary Table S2 and Figure 1.

Focusing on spatial complementarity provided by T1Gado, T2Flair, and FET sequences (Supplementary Table S3), the mean DSC between T1GadoFlair volume and T1GadoFlairFET reached 0.89, highlighting a low spatial difference.

In contrast, the information provided from T2Flair impacted the T1Gado volume since the T1Gado-T1GadoFlair DSC was as low as 0.60 with a standard deviation (SD) of 0.24.

The next stage was to assess the correlation between intra- and inter-sequence variability and PFS/OS.

**Figure 2:**
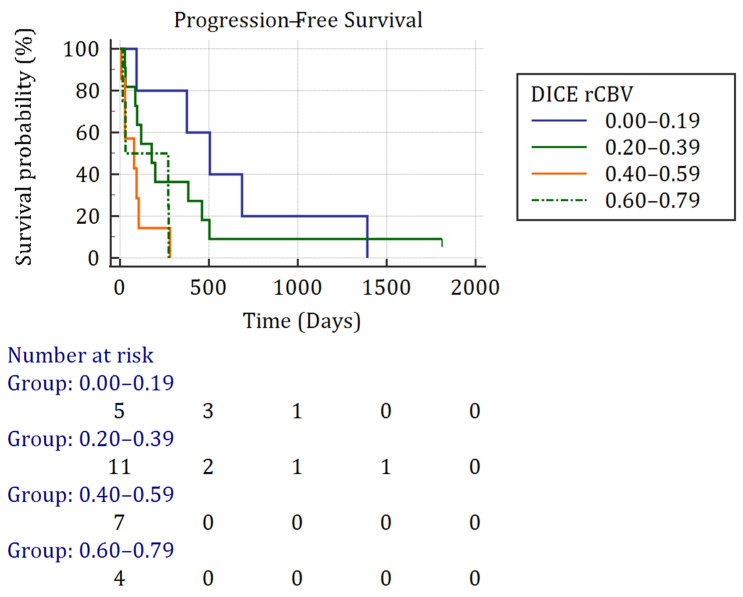
Progression-free survival curve regarding rCBV’s DSC using Kaplan–Meier. Abbreviation: rCBV: relative cerebral blood volume.

Methylation of the MGMT promoter, rCBV variability, rCBV mean volume, and performance of surgery were associated with a *p*-value < 0.20 on the univariate analysis. DSC values ranging from 0.20 to 0.60 (Class 2 and 3) increased the risk of disease progression with respective HR of 12.90 (IC95: 1.43–116.70, *p* = 0.02) and 9.76 (IC95: 1.53–62.13, *p* = 0.02), as shown in Supplementary Table S4. After multivariate analysis, none of the parameters remained significant. As shown in Figure 2, the DSC class had a significant impact on PFS (*p* = 0.02). Surgery lead to a significantly increased PFS (Supplementary Figure S2, *p* < 0.05).

Regarding the prediction of OS, both surgery and rCBV reproducibility were significantly associated with OS. After multivariate analysis, methylation of the MGMT promoter, surgery, and rCBV variability were significantly associated with overall survival (Table 3).

**Figure 3:**
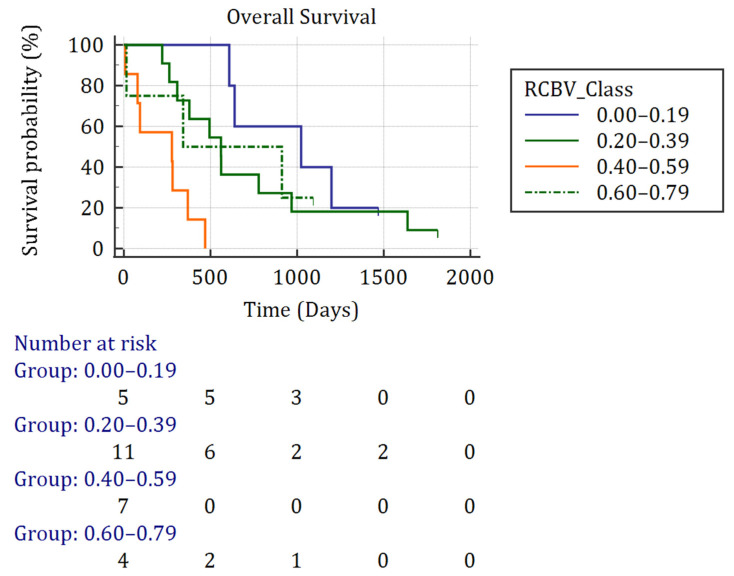
Overall survival curve regarding rCBV’s DSC using Kaplan–Meier. Abbreviation: rCBV: relative cerebral blood volume.

Focusing on rCBV variability, the worst impact on OS concerned the DSC values from classes 2 and 3 with an increased risk of death, HR respectively reaching 28.92 (IC95: 4.47–187.22, *p* < 0.001) and 4.25 (IC95: 0.72–25.1, *p* = 0.11). OS decreased with the agreement on rCBV delineation (Figure 3, *p* = 0.002), while performance of surgery improved OS (Supplementary Figure S3, *p* = 0.0007).

## 4. Discussion

Apart from the quite high reproducibility of T1Gado, T2Flair, and 18 F-FET PET/CT delineations, this study exposes a prognosis value on OS and PFS of rCBV. However, a high variability was shown for rCBV sequences, also highlighting the need for specific training.

Due to its highly vascular and rapidly proliferating cellular characteristics, treating HGGs poses a complex challenge in oncology, even with advanced modern technology. Currently, MRI imaging is considered as the gold standard for HGG radiation treatment planning. However, CRT based only on MRI could possibly undervalue the extent [30].

The use of all available information and especially multimodal imaging [31] has become a rule in oncology, the hope being that the most complete characterization would allow better results. Indeed, more accurate and precise target delineation guidelines for HGGs should help promote standardization and uniformity. Nevertheless, the true value of each sequence, as well as their complementarity and reproducibility, needed further evaluation [32].

Our research focused on the reproducibility of HGG delineations using mpMRI and 18 F-FET PET/CT. The study results suggest that volumes defined from the morphological MRI sequences T1Gado and T2Flair sequences are the most reproducible, followed by 18 F-FET PET/CT, supporting previously published findings that mainly used radiologists’ segmentations [15].

In contrast, lesion volumes defined using functional sequences such as rCBV, k2, ADC, and b1000 from DWI were less reproducible between readers. Our main hypothesis would be the small tumor volumes for these sequences, which could increase the discrepancy between the different physicians due to a more delicate interpretation. Moreover, and by definition, the DSC rapidly decreases for smaller volumes.

Results of poor reproducibility in delineating tumor volumes based on DWI sequences were explained by the fact that DWI is a fast sequence, prone to magnetic susceptibility artifacts (hemorrhage, bone, or sinus proximity), with a low spatial resolution [33]. Exploring the potential impact of shared training in tumor volume delineation using functional MRI sequences on enhancing reproducibility among readers could be interesting, as suggested by Li et al. [33]. Biological imaging was shown to improve tumor detection and radiation treatment planning. The introduction of a biological tumor volume based on biological imaging techniques could lead to a superior tumor coverage [34]. Data on the additional use of amino acid, such as 18 F-FET PET/CT, illustrate a considerably higher specificity of 75–90%, owing to this specific tracer uptake in tumor cells [35,36]. Furthermore, 18 F-FET PET/CT has been shown to be useful in the detection of diffuse glioma infiltration, as described by Verburg et al. [37]. Based on a comparison between histopathology and multimodal imaging, the study showed strong performance in detecting the infiltration of enhanced glioma [37] and the aggressivity by using the difference in uptake of radiotracer, which is correlated with grade and cellularity of HGGs [38]. The target volume delineation definition using amino acid PET is superior to the standard MRI and its inclusion in the surgical planning demonstrated a positive impact on survival [39] with several studies investigating its use for treatment [34,40]. A previous study focusing on the different volume obtained by delineating HGG lesions using MRI and nuclear imaging sequences including 18 F-FET PET/CT [41] highlighted a difference between volumes obtained by MRI and 18 F-FET PET/CT. The 18F-FET PET/CT tumor volume was significantly larger than the T1Gado volume, whereas the hyperintense areas on T2Flair images were larger than both the T1Gado areas and FET uptake areas [41]. In our study, the high DSC suggests that 18 F-FET PET/CT volumes locally correlate with the T1Gado and T2Flair MRI volumes.

PWI provides information on brain hemodynamics through important parameters including rCBV, which is strongly correlated with neoangiogenesis density on histopathology [42]. The rCBV value is calculated by deconvolution of an arterial input function and divided by the value obtained from the normal white matter of the contralateral hemisphere and reflection of the tumor vasculature [43]. The rCBV is not only a validated perfusion parameter to predict tumor grade [43,44] but also a predictive parameter for survival outcomes in patients with HGGs [45]. Increased rCBV values have been reported to extend beyond regions of T1 contrast enhancement with a frequency up to 50%, suggesting a key role for identifying tumor invasion and obtaining a more precise definition of RT planning [46]. Focusing on the repeatability and reproducibility of rCBV measurements in recurrent glial lesions [47], Smits et al. also reported a significant variability in rCBV measurements. Impact of rCBV variability on PFS or OS was not evaluated. In our study, the rCBV variability was predictive for both PFS and OS, meaning that patients with a low rCBV DSC had a longer PFS and OS and vice versa. We hypothesize that these astonishing results could be explained by the correlation between rCBV and tumor vascularization. A higher rCBV DSC is reached for more visible tumors (i.e., highly vascularized tumors) on the rCBV sequence. Given the correlation between the rCBV and tumor aggressiveness [45], we could conclude that the more easily delineated lesions are the most visible on the rCBV sequence and, thus, the most aggressive, leading to shorter survivals. This is the first study describing this supposed correlation; additional studies on a larger cohort should be carried out to confirm this hypothesis.

The higher definition provided by multimodal imaging opens the path for new contouring guidelines with several teams optimizing the treatment by reducing peritumoral margins. This debate remains ongoing with the historic 2 cm margin being recently challenged. Minetti et al. revealed similar patterns of tumor recurrence using a 1 cm GTV-to-CTV (clinical target volume) margin compared with standard target delineation, with a potential benefit of limiting a high radiation dose to normal brain and hippocampi [48]. Regarding Piroth et al., a CTV based on FET with an extra 7 mm may cover 100% of recurrences, leading to reduced PTVs compared to MRI-based PTVs [49]. Of note, the methodology for tumor delineation on PET images differs among studies. The contributions and impacts of molecular imaging are thus difficult to establish. A future study will aim to challenge the PET as a new strategy for delineating the target volumes in patients with recurrent HGGs [50].

To this day, guidelines recommend an isotropic expansion based on the macroscopic tumor itself. However, isotropic margins probably do not fully reflect the complexity of tumor infiltration. Combining multimodal imaging could be a better way to evaluate tumor infiltration and lead to rethinking isotropic expansions.

To address the high local failure rate, there have been several trials evaluating radiation dose escalation, with no clear clinical survival impact [51,52]. The fact that HGGs are inherently infiltrating neoplasms could explain this propensity for local failure, as suggested by the authors. As previously discussed, another reason for the poor results of those studies may be the inability of current imaging methods to adequately reflect the true extent of the tumors [53]. Dose-escalation studies on HGGs used conventional MRI sequences for radiotherapy planning, which could possibly explain the disappointing results, since these sequences provide partial information on tumor extension. Therefore, it is crucial to evaluate localized dose-escalation strategies on more precise imaging modalities, such as functional MRI sequences or biological imaging. If FET uptake translates into a higher rate of recurrence locations, its viability directly influences tumor control probability. Furthermore, in this case, it may be advantageous to administer a simultaneous integrated boost (SIB) to the biological tumor volume/FET-positive regions [53]. A recent dose-painting phase III multicenter in newly diagnosed glioblastoma, where the integrated boost will be guided by MR spectroscopic imaging, despite not leading to increased survivals definitely highlights the interest in this subject [18]. Our results have a direct clinical implication. Only a single study has used the rCBV sequence for target definition in a dose-escalation approach [54]. Given the survival impact of rCBV inter-reader variability in our study, using rCBV for dose escalation should definitely be studied. Doing so will require quality assurance, given the high delineation variability.

The lack of consistency in an interobserver variability study’s methodology was highlighted in a recent literature review, leading to a proposal of the requirement to respect [55]. Our study respects some of them as the STAPLE algorithm was used as a gold standard for the panel of experts. Similarly, volumes were considered to be reproducible when the DSC threshold > 0.7 was reached [55].

However, the following limitations need to be acknowledged: first, it was a monocentric study with a small cohort (N = 30), among which only 27/30 patients had a complete analyzable mpMRI, which may limit the generalizability of the results and could be responsible of the large value extent of rCBV. The data were collected prospectively but delineations were performed retrospectively. Finally, mpMRI was performed on different MRI scanners (27/30 patients on a 1.5 T), which could lead to a certain heterogeneity while increasing the external validity.

## 5. Conclusions

In conclusion, this study confirms the morphological MRI sequences as the most reproducible sequences for the treatment planning of glioblastoma, followed by 18 F-FET PET/CT. Functional sequences bring primordial information on tumor aggressiveness that should not be ignored. A dose escalation on these areas of resistance deserves to be studied, our study having shown a potential impact of rCBV reproducibility on OS and PFS. Implementation of contouring guidelines using multiparametric MRI and possibly 18 F-FET PET/CT for treatment planning of glioblastoma in the daily practice of radiation oncology is critical in an era of decreased target volume expansions and dose painting.

## Figures and Tables

**Table 1:** Baseline characteristics.

Characteristics	Median or n	IQR or Percentage
Age	63	50–69
Sex		
Female	10	33
Male	20	67
PS		
0–1	27	90
2	3	10
Histologic grade (OMS 2017)		
III	5	16.7
IV	25	83.3
MGMT methylation		54.8%
IDH1 or IHD2 mutation		3.3%
Extent of resection		
Biopsy only	14	46.7
Partial (>5% remaining)	4	13.3
Subtotal (<5% remaining)	4	13.3
Complete	8	26.7
Delay between surgery/biopsy and radiotherapy planning CT (days)	22	13–72

Note: data are median (IQR: InterQuartile Range) or n (%), PS: performance status, MGMT: O^6^-methylguanine-DNA-methyltransferase, IDH: isocitrate dehydrogenase, CT: computed tomography.

**Table 2:** Spatial correlation: DSC (mean +/− SD) for each sequence.

	All Pair Readers		MVR		STAPLE	
	Dice Mean	SD	Dice Mean	SD	Dice Mean	SD
T1Gado	0.84	0.09	0.89	0.06	0.89	0.06
T2Flair	0.71	0.18	0.8	0.14	0.8	0.14
K2	0.55	0.18	0.67	0.16	0.68	0.13
rCBV	0.37	0.2	0.53	0.18	0.54	0.16
ADC	0.28	0.17	0.43	0.21	0.51	0.14
Diffusion	0.36	0.12	0.44	0.2	0.56	0.1
FET	0.75	0.11	0.84	0.08	0.82	0.09
T1GadoFlair	0.78	0.13	0.85	0.09	0.84	0.1
T1GadoFlairFET	0.79	0.09	0.86	0.06	0.86	0.06

Note: Dice mean outcomes resulting from comparison of all pairs of readers delineation and from comparison of each reader delineation with majority voting rule (MVR) or STAPLE segmentation.

**Table 3:** Correlation between overall survival (OS) and intra-sequence variability: univariate and multivariable analysis using Cox regression.

		Univariate		Multivariate			
		*p*	HR	*p*	HR	CI95% HR	
						Lower	Higher
Age At Diagnosis		0.21	1.02				
PS		0.37					
Grade		0.81	1.16				
Surgery (ref = no surgery)		0.001	0.26	0.001	0.14	0.05	0.43
IDH (ref = not mutated IDH status)		0.37	0.04				
MGMT (ref = not methylated MGMT)		0.12	0.53	0.04	0.36	0.14	0.94
T1GADO		0.93	0.96				
T2FLAIR (ref = class 1)		0.83					
	Class 2	0.41	0.4				
	Class 3	0.7	0.8				
	Class 4	0.52	0.68				
ADC (ref = class 0)		0.83					
	Class 1	0.90	1.06				
	Class 2	0.46	0.56				
	Class 3	0.69	0.73				
K2 (ref = class 0)		0.86					
	Class 1	0.93	0.91				
	Class 2	0.91	0.89				
	Class 3	0.81	1.29				
	Class 4	0.52	2.53				
RCBV (ref = Class 0)		0.009		0.003			
	Class 1	0.32	1.82	0.15	2.76	0.70	10.93
	Class 2	0.002	10.4	<0.001	28.92	4.47	187.22
	Class 3	0.48	1.73	0.11	4.25	0.72	25.10
Diffusion (ref = Class 0)		0.21					
	Class 1	0.65	1.33				
	Class 2	0.25	0.41				
	Class 3	0.65	1.53				
T1GADOFLAIR (ref = Class 2)		0.95					
	Class 3	0.83	1.15				
	Class 4	0.98	1.02				
T1GADOFLAIRFET (ref = Class 2)		0.75					
	Class 3	0.45	0.55				
	Class 4	0.51	0.6				
FET (ref = Class 2)		1					
	Class 3	0.95	1.04				
	Class 4	0.94	1.05				
Vol_RCBV		0.34	1.01				

Note: Class (0): 0.00–0.19, Class (1): 0.20–0.39, Class (2): 0.40–0.59, Class (3): 0.60–0.79, Class (4): 0.80–1. Abbreviations: PS: performance status, HR: hazard ratio, CI: confidence interval, MGMT: O^6^-methylguanine-DNA-methyltransferase, IDH: isocitrate dehydrogenase.

## Data Availability

Data can be accessed after specific agreement from the authors and the ethical committee.

## Supplementary Materials

The following supporting information can be downloaded at https://www.mdpi.com/article/10.3390/biomedicines12040789/s1, Table S1: Main MRI acquisition parameters; Table S2: Dice comparison (*p*-value) between each sequence (Wilcoxon test); Table S3: Spatial complementarity: DSC (mean +/− SD) for T1Gado, T2Flair and FET; Table S4: Correlation between progression free survival (PFS) and intra-sequence variability: univariate and multivariable analysis using Cox regression; Figure S1: Progression-Free Survival curve regarding surgery using Kaplan Meier; Figure S2: Overall Survival curve regarding surgery using Kaplan Meier.
