# Supplementary material for: Correlation between rCBV Delineation Similarity and Overall Survival in a Prospective Cohort of High-Grade Gliomas Patients: The Hidden Value of Multimodal MRI?
Source: Biomedicines. 2024 Apr 3;12(4):789. doi: 10.3390/biomedicines12040789 (PMC11048661; doi:10.3390/biomedicines12040789)

SUPPLEMENTARY MATERIALS

Supplementary Table S1. Main MRI acquisition parameters

Supplementary Table S2. Dice comparison (p-value) between each sequence (Wilcoxon test).

Supplementary Table S3. Spatial complementarity: DSC (mean +/- SD) for T1Gado, T2Flair and FET.

Supplementary Table S4. Correlation between progression free survival (PFS) and intra-sequence variability: univariate and multivariable analysis using Cox regression.

Supplementary Figure S1. Progression-Free Survival curve regarding surgery using Kaplan Meier.

Supplementary Figure S2. Overall Survival curve regarding surgery using Kaplan Meier.

Supplementary Table S1. Main MRI acquisition parameters

MRI scanner	SIEMENS 1.5 T				GE MEDICAL SYSTEM 1.5			
SEQUENCE	TE (ms)	TR (ms)	MATRIX	SLICE THINKNESS (mm)	TE (ms)	TR (ms)	MATRIX	SLICE THINKNESS
T1 GADOLINIUM	7.1	600	256 x 256	1	16.74	452	256 x 256	1
T2 FLAIR	335	5000	256 x 217	1	137	452	192 x 192	1,6
rCBV	30	1650	128 x 128	5	30	2290	128 x 128	5
DIFFUSION/ADC	95	2800	192 x 192	4	69.7	9645	128 x 128	4
K2	30	1650	128 x 128	5	30	2290	128 x 128	5

Abbreviations: MRI: Magnetic Resonnance Imaging, TE: Time of Echo, TR: Time of Repetition, ms: mili-second, rCBV: Relative cerebral blood volume, ADC: Apparent diffusion coefficient

Supplementary Table S2. Dice comparison (p-value) between each sequence (Wilcoxon test).

DICE Comparison	T1Gado	T2Flair	T1GadoFlair	T1GadoFlairFET	K2	rCBV	ADC	Diffusion
**T1Gado**	-							
**T2Flair**	< 0.0001	-						
**T1GadoFlair**	0.0001	< 0.0001	-					
**T1GadoFlairFET**	0.0005	0.0004	0.69	-				
**K2**	< 0.0001	0.0013	0.0001	< 0.0001	-			
**rCBV**	< 0.0001	< 0.0001	< 0.0001	< 0.0001	0.0001	-		
**ADC**	< 0.0001	< 0.0001	< 0.0001	< 0.0001	< 0.0001	0.01	-	
**Diffusion**	< 0.0001	< 0.0001	< 0.0001	< 0.0001	0.0003	0.92	0.04	-
**FET**	0.008	0.30	0.32	0.13	0.0001	< 0.0001	< 0.0001	< 0.0001

Supplementary Table S3. Spatial complementarity: DSC (mean +/- SD) for T1Gado, T2Flair and FET.

	DICE MEAN (STANDARD DEVIATION)			
**SEQUENCE**	**T1GADO**	**T2FLAIR**	**FET**	**T1GadoFlair**
**T1GadoFlair**	0.60 (0.24)	0.91 (0.16)	0.45 (0.17)	-
**T1GadoFlairFET**	0.52 (0.21)	0.82 (0.21)	0.59 (0.20)	0.89 (0.14)

Supplementary Table S4. Correlation between progression free survival (PFS) and intra-sequence variability: univariate and multivariable analysis using Cox regression.

Note. Class (0): 0.00-0.19, Class (1): 0.20-0.39, Class (2): 0.40-0.59, Class (3): 0.60-0.79, Class (4): 0.80-1

Multivariate analysis was conducted but only with preselected significant ($p < 0.20$) variables.

PS: performance status, HR: Hazard Ratio, CI: Confidence Interval, MGMT: O^6-methylguanine-DNA-methyltransferase, IDH : isocitrate deshydrogenase

		Univariate		Multivariate			
		p	HR	p	HR	CI95% HR	
						Lower	Higher
AGE *		0.97	1				
PS		0.43					
GRADE		0.54	1.40				
SURGERY (ref = no surgery)		0.05	0.47	0.06	0.40	0.15	1.02
IDH (ref = not mutated IDH status		0.22	0.28				
MGMT (ref = not methylated MGMT)		0.05	0.47	0.08	0.43	0.16	1.12
T1GADO		0.25	1.6				
T2FLAIR (ref = class 1)		0.73					
	Class 2	0.5	0.54				
	Class 3	0.71	1.25				
	Class 4	0.81	0.87				
ADC (ref = class 0)		0.69					
	Class 1	0.87	0.94				
	Class 2	0.25	0.45				
	Class 3	0.82	0.84				
K2 (ref = class 0)		0.58					
	Class 1	0.5	2.13				
	Class 2	0.4	2.46				
	Class 3	0.29	3.05				
	Class 4	0.12	9.96				
RCBV (ref = Class 0)		0.04		0.09			
	Class 1	0.28	1.83	0.13	2.55	0.75	8.66
	Class 2	0.01	6.17	0.02	12.90	1.43	116.70
	Class 3	0.05	4.44	0.02	9.76	1.53	62.13
DIFFUSION (ref = Class 0)		0.26					
	Class 1	0.65	0.59				
	Class 2	0.73	0.36				
	Class 3	0.95	0.98				
T1GADOFLAIR (ref = Class 2)		0.58					
	Class 3	0.3	1.88				
	Class 4	0.5	1.46				
T1GADOFLAIRFET (ref = Class 2)		0.96					
	Class 3	0.93	1.07				
	Class 4	0.84	1.17				
FET (ref = Class 2)		1					
	Class 3	0.92	1.06				
	Class 4	0.92	1.07				
Vol_RCBV		0.12	1.01	0.29	0.98	0.95	1.02

Supplementary Figure S1. Progression-Free Survival curve regarding surgery using Kaplan Meier.

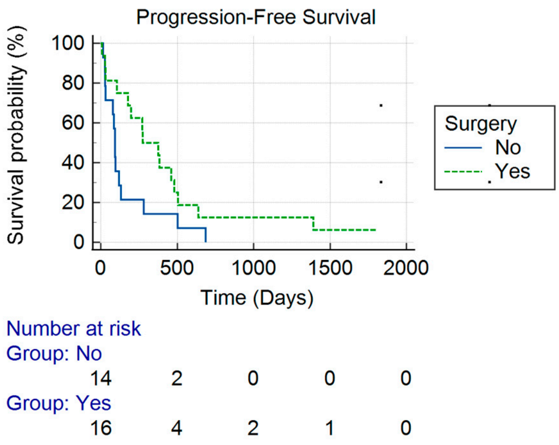

Supplementary Figure S2. Overall Survival curve regarding surgery using Kaplan Meier.
Time is expressed in days

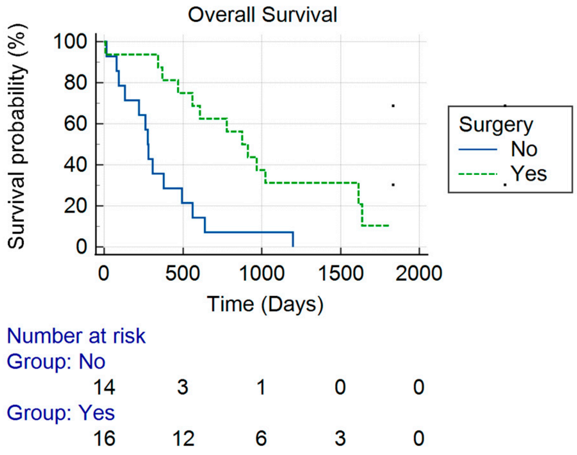

Supplement: Supplementary file 1 [file biomedicines-12-00789-s001.zip › biomedicines-2921703-supplementary.pdf]
